# Unmet needs in patients with RA judged in remission by the rheumatologist: a semi-structured interview study

**DOI:** 10.1093/rap/rkag023

**Published:** 2026-02-17

**Authors:** Elias De Meyst, Marine Piessens, Jeannine Engelen, Delphine Bertrand, Michaël Doumen, Nicolas Degryse, Nele Verbeuren, René Westhovens, Patrick Verschueren

**Affiliations:** Department of Development and Regeneration, KU Leuven, Leuven, Belgium; Department of Rheumatology, University Hospitals Leuven, Leuven, Belgium; Department of Rheumatology, University Hospitals Leuven, Leuven, Belgium; RA Liga vzw, Herentals, Belgium; Department of Development and Regeneration, KU Leuven, Leuven, Belgium; The Greenhouse, AZ Groeninge, Kortrijk, Belgium; Department of Development and Regeneration, KU Leuven, Leuven, Belgium; Department of Rheumatology, University Hospitals Leuven, Leuven, Belgium; Department of Development and Regeneration, KU Leuven, Leuven, Belgium; Department of Rheumatology, University Hospitals Leuven, Leuven, Belgium; Department of Development and Regeneration, KU Leuven, Leuven, Belgium; Department of Rheumatology, University Hospitals Leuven, Leuven, Belgium; Department of Development and Regeneration, KU Leuven, Leuven, Belgium; Department of Rheumatology, University Hospitals Leuven, Leuven, Belgium; Department of Development and Regeneration, KU Leuven, Leuven, Belgium; Department of Rheumatology, University Hospitals Leuven, Leuven, Belgium

**Keywords:** RA, qualitative research, disease burden, disease impact, patient perspective

## Abstract

**Objectives:**

To map unmet care needs in patients with RA who are in remission, investigate perceptions on the cause and impact of such needs and explore experiences and beliefs about suitable care strategies to address these issues.

**Methods:**

Individual in-depth semi-structured interviews were conducted in patients with RA judged in remission by rheumatologists until data saturation. Qualitative data analysis was performed according to a guide, based on the constant comparative method.

**Results:**

Seventeen patient interviews were included for analysis. Patients identified a variety of persistent symptoms, like fatigue or anxiety, despite being judged in remission, reporting a substantial impact on different life domains. Multiple issues were found to be coexistent, reinforcing each other. Uncertainty thrived in patients regarding the aetiology of their persistent problems, with symptoms often attributed to undetectable inflammation. Furthermore, patients were unsure which additional care strategies could be of help. Often, rheumatologists were perceived to give insufficient attention to the unmet needs of patients. Patients recognized the potential benefits of receiving support from a multidisciplinary team, but identified important barriers limiting engagement with such care, including a lack of knowledge on the team’s availability and competences. Care was preferred to be tailored to individual needs and encouraging.

**Conclusion:**

Our findings emphasize that unmet needs in patients with RA in remission require and deserve additional attention. Although some barriers were identified, patients recognized the potential benefits of receiving multidisciplinary support. In this regard, complementary care should be needs-based, flexible and empowering.

Key Messages

- Persistent non-inflammatory disease burden in patients with RA in remission requires professional support.
- Rheumatologist care is often deemed insufficient, prioritizing disease activity while insufficiently addressing non-inflammatory needs.
- Patients recognize benefits of multidisciplinary care, yet barriers reduce their openness to such support.

## Introduction

Rheumatoid arthritis (RA) is a chronic inflammatory joint disease with a substantial impact on patients’ quality of life, as well as on a socioeconomic level [1]. Fortunately, treatments have evolved over the last decades, and many patients achieve good disease control. Nevertheless, at least one in five patients who are in remission report persistent issues [2–5], indicating unmet needs that are not directly related to active disease. Examples include persistent fatigue, joint pains and functional impairment [6]. To facilitate the identification of unmet needs in patients with RA, several tools have been proposed, including the Rheumatoid Arthritis Impact of Disease instrument (RAID) [7] or consideration of its individual subcomponents [8], the Patient Acceptable Symptom State [9], the Patient Experienced Symptom State (PESS) [10] and a patient-physician discordance score (DS) [11]. Complementary non-pharmacological interventions provided by physiotherapists, psychologists, occupational therapists and other healthcare professionals (HCPs) have been shown to alleviate symptomatic burden in RA and improve health outcomes [12–14]. Furthermore, the role of advanced practice nurses (APNs) is well-established in rheumatology clinics, and nurse-led care models have been proven to be equally effective in terms of health outcomes and to be cost-effective compared with rheumatologist-led care, even excelling in outcomes like patient satisfaction [15–20]. APNs may play key roles in offering self-management support and coordinating interdisciplinary care. In Belgium, however, a legal framework for the recognition of the APN title was only recently established [21], implicating that only few patients are familiar with this HCP.

An important question that remains is how patients with RA in remission perceive interventions from HCPs other than their rheumatologist, and how this perception influences their receptivity to multidisciplinary support. Beliefs may be driven by the nature and impact of issues, ideas about the role of HCPs, previous experiences with healthcare and so on. In addition, illness perceptions are crucial to consider as they correlate with outcomes like pain and fatigue in RA and other rheumatic conditions [3, 22, 23]. In this qualitative study, we aimed to map persistent needs in patients with well-controlled RA, examine their impact and uncover patient perceptions about the aetiology of such issues. In addition, we explored patient experiences and beliefs about suitable management strategies to address their problems, with a particular interest in views on multidisciplinary care.

## Methods

### Study design and population

Individual semi-structured interviews were conducted in patients with RA. Patients were recruited from the outpatient rheumatology clinic at a tertiary centre in Leuven, Belgium. Patients were eligible for inclusion if they were ≥18 years old, were diagnosed with RA more than 1 year ago, were fluent in Dutch, were in clinical remission judged by a rheumatologist and were suspected of having unmet needs, based on a positive DS ≥0.15 at the previous visit at the clinic, which could have taken place up to 6 months before the screening visit. The DS balances patient-reported outcomes (pain, functional impairment, fatigue and the patient’s assessment of disease activity) with traditional disease activity measures (tender and swollen joint counts, the rheumatologist’s assessment of disease activity and laboratory markers of inflammation) [11]. The resulting score ranges from −1 to +1, with positive scores indicating higher patient-reported burden relative to the burden reported by the rheumatologist and the laboratory assessment, indicating a potential presence of unsolved issues. Aiming to capture a broad range of perspectives, patients were recruited using purposive sampling based on several sociodemographic and disease-related characteristics, including age, sex, time since diagnosis, employment status, family situation and education level. All patients gave written informed consent. The study was approved by the Ethics Committee Research UZ/KU Leuven (S68348) and is reported according to the Consolidated Criteria for Reporting Qualitative Research checklist (Supplementary Data S1, available at *Rheumatology Advances in Practice* online) [24].

### Interview guide

The interview guide (Supplementary Data S2, available at *Rheumatology Advances in Practice* online) was designed through rounds of discussion between the members of the research team, including three doctoral researchers active in the field of clinical rheumatology, two expert rheumatologists, one APN and one patient research partner. All members of the research team had previous experience conducting qualitative research. After eight interviews, the interview guide was adapted through further rounds of discussion, in line with qualitative research conduct [25]. The primary aim was to gain better insights into patient experiences and perspectives on the role of the nurse and other HCPs in rheumatology care, which were felt to be incomplete at that moment. In addition, one complementary question probing whether patients felt their assessment of disease status was in line with the rheumatologist’s assessment was deleted, as participants had difficulties understanding the question, and because the complementary question did not seem to result in additional insights. The adapted version of the interview guide can be found in Supplementary Data S3, available at *Rheumatology Advances in Practice* online.

### Data collection and saturation

Interviews were conducted by two researchers (E.D.M. and M.P.) in person or via videocall within 2 weeks of the screening visit, according to patient preferences. A family member was allowed to attend the interview to provide comfort and emotional support, or to help participants recollect previous experiences. As the presence of an additional person may influence participants’ responses, interviews were carefully monitored to minimize interference with the interview process. Prior to every interview, participants completed the RAID questionnaire, a quantitative measure of disease impact [7]. Interviews continued until data saturation, which was defined beforehand as a lack of new insights emerging from the last three interviews. All interviews were audiotaped and transcribed in verbatim, accompanied by field notes. After data analysis, audiotapes were erased.

### Data analysis

A thematic data analysis was conducted in accordance with the Qualitative Analysis Guide of Leuven (QUAGOL), which is based on the principles of grounded theory, implementing the constant comparative method [26]. Two researchers (E.D.M. and M.P.) independently coded the transcribed interviews using NVivo 15 software. Next, emerging codes were refined through rounds of discussion between both researchers. Subsequently, both researchers independently grouped the refined codes into overarching themes and subthemes, which were then repeatedly discussed with the full research team, until agreement resulted in the final thematic analysis.

### Patient involvement

In line with EULAR recommendations [27], one patient research partner (J.E.) was involved throughout the study. She provided feedback on the study protocol, including the interview guide and informed consent forms, with particular attention to clarity for patients and the relevance of the study objectives from a patient perspective. During the data analysis phase, the patient research partner participated in discussions on preliminary themes and subthemes, contributing to the refinement of the final thematic structure and the interpretation of the results.

## Results

### Participants

Between January and June 2024, 18 patients were interviewed. In three interviews, a family member was present. One interview was excluded from analysis as the family member actively steered the interviewee’s answers, influencing the reliability of data. The demographics of the remaining 17 patients can be found in Table 1. The median interview duration was 42 min (range: 14–73 min).

**Table 1:** Participant characteristics.

Characteristics	Patients (*n *= 17)
Age (years)	59 (21–81)
Sex (female)	65% (11/17)
Time since diagnosis (years)	11 (1–41)
Discordance score (−1 to +1)	0.32 (0.17–0.75)
Total RAID score (0-10)	3.7 (0.0–9.8)
Tertiary education (yes)^a^	44% (7/16)
Employed (yes)	59% (10/17)
Having a partner (yes)	76% (13/17)
Having children (yes)	82% (14/17)

Expressed in median (range) or % (n/n).

a For one patient, this information was not available.

RAID: rheumatoid arthritis impact of disease.

### Thematic analysis

**Figure 1:**
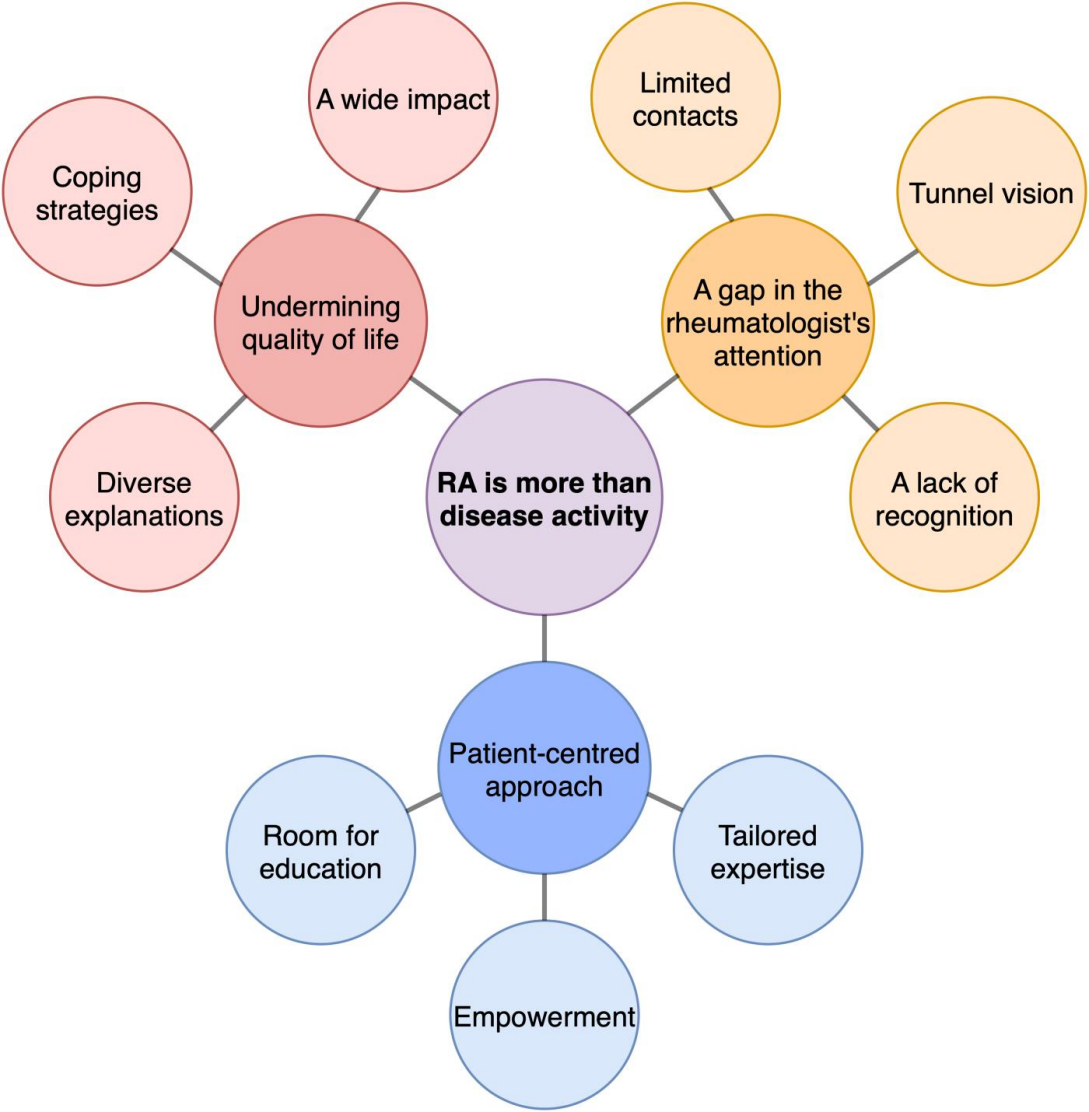
Qualitative data analysis. Three overarching themes were identified, related to the central perception that living with RA goes way further than dealing with joint inflammation. Every overarching theme is substantiated with three corresponding subthemes

Throughout the data analysis, the central perception arose that living with RA involves much more than dealing with inflammatory disease activity. Three overarching themes emerged related to this central perception, namely that persistent unmet needs significantly undermine quality of life, that they get insufficient attention from the rheumatologist and that they require and deserve a patient-centred approach. Every overarching theme was substantiated by three subthemes (Fig. 1).

### Unmet needs undermine the patients’ quality of life

#### A wide impact

Patients reported a variety of ongoing problems, including pain, fatigue, emotional distress, functional impairment and sleeping problems. Emotional distress was expressed in various ways, describing feelings of anxiety, depression, anger, frustration, sadness and loneliness. As a result, a profound impact was voiced:

*[RA] dominates your whole life. And it ruins a lot. You have the right to a good, healthy life, and you don’t have that anymore.*

– Patient 5, female, 77 years

Living with RA was expressed as a daily struggle, being constantly confronted with one’s own limitations. Persistent issues manifested in various life domains, impairing social interactions, intimate relationships, studying, work and hobbies. Often, different issues were found to coexist and reinforce one another, for example, fatigue leading to functional impairment, in part by inciting anxiety:

*Fatigue is super annoying. Because actually, it limits you in what you can and cannot do. Primarily, you don’t do things because you are afraid you will be too tired to do them.*

– Patient 8, male, 62 years

As a result of their struggles, patients expressed feelings of isolation, mourning a loss of spontaneity in life. Furthermore, ongoing issues were associated with the rise of new health problems, for instance gaining weight due to limited physical activity, caused by pain and functional impairment. Although the impact of problems varied individually, a common desire was expressed to maintain autonomy and continue to do things which bring a sense of joy and fulfilment.

#### Diverse explanations

Patients had various explanations for the aetiology of their problems. At many times, patients reported having no idea about the cause of their issues, which was often the case for fatigue. Frequently, explanations were sought in the pathophysiology of the disease. A recurring belief included that problems were caused by undetectable inflammation:

*It’s not always the case that a swelling or inflammation is visible, but I think that there is always something gnawing on the joints.*

– Patient 4, female, 55 years

Other recurrent explanations included suspected or confirmed comorbidities like osteoarthritis or cardiac disease, or side effects of antirheumatic drugs. In contrast, circumstances unrelated to medical conditions were mentioned as well, like stress caused by familial and professional circumstances, financial worries or weather conditions. In addition, age was often mentioned as an explanatory factor. Patients proposed the idea that their unmet needs were caused by a combination of many different factors. They also explained that personality traits and coping style could influence whether something was perceived as an issue or not:

*As a patient, if you have such [a passive] attitude, and your doctor accepts this, yes, then you are a chronic patient. The ‘chronic’ part is mainly in your head, then.*

– Patient 8, male, 62 years

#### Coping strategies

Patients had developed several ways to deal with their problems without having to immediately reach out to the rheumatology clinic, illustrating the diversity of coping strategies. Medication, foremost painkillers and anti-inflammatory drugs, were often mentioned to be helpful, alleviating symptoms of pain or fatigue at least partially. Actively seeking for warm and dry weather, and heat applications were also found to be beneficial. Nutritional supplements were taken to increase energy levels. One patient recommended cryotherapy, provided by a practitioner of alternative medicine. Most patients mentioned the importance of allowing flexibility in their life, as often unforeseen symptoms required professional or household tasks to be postponed or delegated:

*I have the luxury of having a flexible work schedule. (…) I know when there are busy periods. I can sort that out myself a bit and plan when I can rest.*

– Patient 7, female, 54 years

Some patients had switched to jobs that were less physically demanding, easing pain, fatigue and functional limitations. Maintaining a healthy lifestyle was deemed vital, mentioning physical activity and striving for a healthy weight. For emotional distress, some patients found relief by sharing concerns with friends or family. Although self-management interventions could often reduce the impact of persistent problems, a significant disease burden often remained. Many patients had learnt to accept their situation to some degree, which was deemed necessary as part of living with a chronic condition. While most patients took up a combative stance to deal with their problems, some patients believed that nothing could help anymore for improving their situation, expressing feelings of hopelessness and despair. Such thoughts were often linked to the psychosocial context, as well as the perception that RA is a progressive disease that inevitably leads to functional disability:

*You’ll deteriorate with arthritis anyway. Don’t lie, because it’s the truth. I know that well enough. There are plenty of people [in the waiting room] who are in wheelchairs. Even young people.*

– Patient 3, female, 74 years

### Unmet needs receive insufficient attention from rheumatologists

#### Limited points of contact

Although patients were confronted with their issues on a daily basis, opportunities to discuss concerns with rheumatologists were limited in time. Indeed, several patients wished for longer consultations on a more frequent basis. Patients observed a shift in the frequency of consultations throughout the years, as better disease control resulted in fewer contacts:

*In the beginning, you come visit every three months or so. Eventually, you only visit every six months. (…) So, at that moment, it is an important meeting for me, and I want to have everything in order. (…) I have noticed in the last couple of years, I feel like: I only come every six months, so I must write everything down, and I have to ask this and that.*

– Patient 7, female, 54 years

Patients experienced that rheumatologists were hard to reach for advice outside consultations. Furthermore, contacts with the rheumatologist often felt rushed, which was attributed to the rheumatologists’ busy working schedules, or more negative assumptions that rheumatologists prefer not to invest more time in patients. Consequently, unmet needs remained unaddressed. Sometimes, patients were seen by different rheumatologists throughout their follow-up, which was less appreciated, as the same issues had to be explained over and over again, allowing no time for in-depth discussions.

#### Tunnel vision

Several patients felt their rheumatologist was caught by tunnel vision. An overwhelming focus on drug therapy was observed, as well as a lack of attention for symptoms which do not fit the textbook image of uncontrolled RA:

*I have the feeling that [rheumatologists] only focus on rheumatism. For a few years now, I have indicated several times that I suffer from migraines and fatigue, but they only keep on focusing on rheumatism and the treatment of rheumatism.*

– Patient 16, female, 21 years

In line with this observation, patients noticed that rheumatologists were mostly triggered by the appearance of new symptoms, or an acute increase in existing symptoms. Long-standing symptoms that were stable, even when disease burden remained high, were often not considered to be alarming or did not result in any change of management. In this regard, a lack of response to the symptom of fatigue was mentioned repeatedly, a symptom often minimized, normalized (‘everyone suffers from fatigue’) or even ignored. Additionally, when persistent symptoms were brought up, some patients felt they were judged negatively as being attention-seeking or having mental health issues. This sense of prejudice from the rheumatologist’s part was felt to be fed by statistics and results of clinical studies, instead of considering individual circumstances. Another frustration was a lack of additional testing or referral to other medical specialties when symptoms were persistent, which was perceived as a form of negligence.

#### A lack of recognition

Altogether, the gap in the rheumatologist’s attention for persistent needs resulted in patients expressing feelings of not being believed, being misunderstood, or being treated with a lack of respect. Some felt that the impact of persistent problems was systematically underestimated. Views of patients and rheumatologists on disease status were often misaligned, putting strain on the therapeutic relationship. The experience that certain long-existing symptoms were inadequately addressed even led to doubts regarding the rheumatologists’ professional competences. In line with this, the usefulness of follow-up visits was sometimes questioned:

*It sometimes just feels like a compulsory visit every three or six months. If I don’t see change in the situation, if it stays the same, then it sometimes feels like a compulsory visit. Like, okay, we’ll just go [to the clinic] again, knowing pretty much beforehand that if I do not have new symptoms, then the situation will stay the same. Then nothing else is discussed anymore, or the treatment stays the same, and that’s it.*

– Patient 14, male, 43 years

### Unmet needs deserve a patient-centred approach

#### Room for education

Patients expressed a lack of knowledge on several regards, desiring additional education. First, it was often unclear to patients whether persistent symptoms were related to active RA or not. The observation that rheumatologists did not always explain the difference in aetiology of symptoms gave rise to uncertainty: patients worried whether their RA was insufficiently controlled, or that they were suffering from potentially dangerous undiagnosed conditions. Second, often questions lingered on how to positively influence RA through lifestyle changes: patients wanted advice on foods to avoid, and whether it was safe to practise sports when still experiencing joint pain. Third, although limitations of antirheumatic drugs in terms of symptom relief were recognized, many patients were unaware that multidisciplinary support was available. Furthermore, misconceptions were thriving on the role of different HCPs:

*(About the occupational therapist) What kind of animal is that?*

– Patient 13, male, 62 years

*What could [nurses] do for me? I can inject my medication myself.*

– Patient 11, male, 59 years

#### Empowerment

When asked to describe what type of care they needed, patients expressed a desire for empowerment. Patients disclosed past experiences in rheumatology clinics, warning about the detrimental effects of a patronizing approach or a focus on the negative consequences of RA, provoking anxiety and guilt. Instead, patients needed hope and positive prospects:

*It is very important that there is still some perspective. You have to know that there still is a way forward, that it doesn’t have to be a downward spiral.*

– Patient 8, male, 62 years

To deal with the ongoing struggles associated with RA, most patients agreed that HCPs should use a motivational and activating approach, inspiring a combative stance and positive mindset.

#### Tailored expertise

To deal with complex unmet needs, patients stated they needed additional expertise. Although the ultimate responsibility of care was placed on the rheumatologist, foremost patients who had previous experience with multidisciplinary approaches proclaimed a need for adjuvant care strategies offered by different HCPs, talking about nurses, physiotherapists, psychologists, occupational therapists, dieticians and social workers. Patients believed that multidisciplinary care could significantly improve the quality of care. Another perceived benefit included having more time to discuss concerns with professionals. Sometimes, being able to voice concerns to a professional and being listened to resulted in a sense of relief, without any further actions. Furthermore, the presence of a multidisciplinary team was seen as a way of having easier access to the rheumatology clinic, and in particular rheumatologists themselves:

*I have a physiotherapist who really knows the disease, who knows my medical files, who can also go directly to the nurse, and who can immediately meet up with the rheumatologist.*

– Patient 17, female, 31 years

However, not all patients expressed an openness towards multidisciplinary interventions, as several barriers were identified. Patients brought up practical concerns, including time investments, costs and long travel distances to the hospital to receive additional care. Some patients simply did not believe in the benefits of multidisciplinary interventions, or found certain types of care to be stigmatizing, like being seen by a psychologist, or receiving advice about sexual health. Others stated that additional contacts would mean repeated confrontations with RA and with the healthcare environment, which was perceived negatively:

*Yes, you also have to be willing to accept [additional support]. For the time being, I don’t really see much need for myself to see other healthcare providers. (…) If you accept more help or see more care providers, you are constantly reminded of your problems.*

– Patient 14, male, 43 years

Others expressed concern that additional care might lead to the identification of new problems, which could give rise to anxiety. Either way, most agreed that referral to other HCPs should be discussed with patients, considering individual needs and preferences:

*You have to understand that every patient is different. Every patient has different symptoms, like pain. The disease is different in every patient. (…) I have seen patients who said they were completely fine, that they didn’t need anything extra. They just went to the hospital and followed the drug treatments. They could literally carry on with everything as normal. But that wasn’t my situation.*

– Patient 17, female, 31 years

Most patients expected the rheumatologist to discuss the availability of multidisciplinary interventions, while others felt different actors could take up this role, referring to nurses or psychologists. Patients agreed that multidisciplinary care should be flexible: different needs require different care approaches, and systematic follow-up by other HCPs may not always be necessary. One patient believed that complementary care should be gradually introduced and discussed with all patients once inflammatory disease control is achieved, at least to some extent, by antirheumatic drug therapy. Finally, patients expressed that needs and expectations evolve through a lifetime, implying that continuous discussions are necessary to adjust care.

## Discussion

This study confirmed the presence of various persistent issues in patients with RA judged in remission, but with a discordance between their own assessment of disease control and traditional clinical and laboratory parameters. Many patients still attributed their struggles to undetectable inflammatory disease. Nonetheless, patients often perceived the cause of symptoms to be multifactorial, seeking explanations within a biopsychosocial context. Patients perceived a lack of attention for unmet needs from rheumatologists, provoking negative thoughts on the rheumatologists’ competences and good will. To deal with struggles, patients expressed a desire for personalized, empowering and flexible care, hoping to maintain their autonomy. Although the benefits of multidisciplinary care were identified, receptibility was limited by several barriers, including a lack of knowledge on the availability and qualifications of HCPs other than rheumatologists.

In line with our findings, previous qualitative research has demonstrated that patients with RA perceive a lack of attention from the rheumatologist’s part regarding non-pharmacological approaches, leading to frustration and disappointment [28]. Although maintaining adequate disease control with antirheumatic drug therapies remains the foundation of RA treatment, care should include an aim to relieve disease burden regardless of inflammatory status. In this regard, and in accordance with other studies [29, 30], we demonstrate that patients desire multidimensional support, considering this to be part of high-quality care. Patients expressed a need to be activated and motivated to exert greater control over their situation, highlighting empowerment as a key component of care. Patient empowerment is central to effective self-management and meaningful participation in shared decision-making and has been associated with higher patient satisfaction and improved health outcomes [31, 32]. Multidisciplinary support should be tailored to individual needs, avoiding a one-size-fits-all approach [33], in accordance with views expressed in the present study. As needs and expectations were recognized to evolve through a lifetime, true tailored care should include repeated discussions to reconsider patients’ needs. However, barriers limiting engagement with other HCPs should be addressed. Concerns about costs should be mitigated. Furthermore, patients feared the time investment necessary to receive additional care, especially considering the travel distance to the clinic. Involving general practitioners, primary care nurses and allied HCPs may mitigate this concern, providing care closer to home [34]. Other barriers included a lack of belief in the expertise of HCPs, which was often driven by previous negative experiences, or misconceptions about the role of HCPs. This could at least in part be mitigated by patient education.

Interestingly, patients in this study indicated that persistent issues like pain, fatigue, emotional distress and functional impairment were often found to coexist, reinforcing one another. Thus, a comprehensive approach seems necessary to deal with different struggles, but addressing one particularly prominent need may also result in the alleviation of other problems to some extent. Therefore, patients should be encouraged to prioritize the needs they want to see addressed, and goal-setting could facilitate this process [31].

At a time when rheumatologists experience significant work burden, APNs could take up key roles in addressing persistent needs in patients with RA. First and foremost, by improving the dialogue with patients, they could help identify important persistent problems. As some topics might not be effectively brought to the attention of HCPs by patients, the use of screening tools like the DS or PESS might be helpful to identify persistent issues [10, 11]. Second, APNs could educate patients on the identified needs in terms of aetiology and provide self-management support. Third, APNs could identify, discuss and refer patients who might benefit from interventions provided by other HCPs part of the multidisciplinary team through shared decision-making, thus acting as a key coordinator of interdisciplinary care. As healthcare resources are increasingly put under pressure, implementing novel holistic healthcare models remains challenging. Although governmental policies are changing, ongoing efforts are required in certain countries to create the legal frameworks to ensure proper education and remuneration of HCPs, while not making patients bear excessive costs.

This study had some limitations. To investigate perceptions on persistent needs not directly related to active inflammation, patients were selected based on the rheumatologist’s clinical judgement of remission rather than using disease activity measures, allowing a margin of error. Composite disease activity measures, however, have similar important limitations, as they include components that may reflect disease burden rather than disease activity [35]. In addition, selection of patients with a suspicion for the presence of unmet needs was based on the DS calculated at the previous visit at the clinic, which could take place 6 months earlier at the most, a period in which needs may change. The reason we used the DS of the previous clinic visit was purely practical, given that the DS incorporates laboratory parameters of inflammation which were not readily available at the screening visit. Furthermore, all patients were recruited from one tertiary centre in Belgium, which might limit transferability of our findings to other settings, especially considering international differences in healthcare policy, which surely influence perceptions on the role of HCPs in rheumatology care. In addition, we only interviewed patients who were fluent in Dutch for practical reasons, therefore potentially missing perceptions of patients from different cultural backgrounds, who may present with their own specific needs. Nonetheless, patients were invited based on purposive sampling to aim for a diverse sample.

Our study had several strengths. First, the interview guide was designed in collaboration with an interdisciplinary research team, and the guide was reevaluated and refined throughout the study conduct to enhance the relevance of the data. Second, the data analysis was performed in line with the QUAGOL, a qualitative analysis guide, via an inductive, data-driven approach without a predefined framework. Third, the coding process and data analyses were performed by two independent researchers with different backgrounds (one physician and one APN), ensuring the robustness of findings, while the final thematic analysis was the result of several rounds of discussions with the interdisciplinary research team, including a patient research partner.

## Conclusion

This study emphasizes that unmet needs in patients with RA in remission require and deserve additional attention which may escape the rheumatologist’s focus. Patients wished for education on the aetiology and potential management strategies to deal with their persistent issues. Although some barriers were identified, patients perceived the potential benefits of receiving multidisciplinary support. In this regard, complementary care should be needs-based, flexible and empowering.

## Supplementary Material

rkag023_Supplementary_Data

## Data Availability

Raw data are available from the corresponding author upon reasonable request.
