# Supplementary material for: Unmet needs in patients with RA judged in remission by the rheumatologist: a semi-structured interview study
Source: Rheumatol Adv Pract. 2026 Feb 17;10(2):rkag023. doi: 10.1093/rap/rkag023 (PMC13006061; doi:10.1093/rap/rkag023)
Supplement: rkag023_Supplementary_Data [file rkag023_supplementary_data.docx]

**Supplementary Data S1:**

**Consolidated criteria for reporting qualitative studies (COREQ): 32-item checklist**

| **No. Item** | **Guide questions/description** | **Reported on Page #** |
| --- | --- | --- |
| **Domain 1: Research team and reﬂexivity** |  |  |
| *Personal Characteristics* |  |  |
| 1. Interviewer/facilitator | Which author(s) conducted the interview or focus group? | Page 6 |
| 2. Credentials | What were the researcher’s credentials? E.g. PhD, MD | Page 17 |
| 3. Occupation | What was their occupation at the time of the study? | Page 17 |
| 4. Gender | Was the researcher male or female? | N/A (EDM is male, MP is female) |
| 5. Experience and training | What experience or training did the researcher have? | Page 5 |
| *Relationship with participants* |  |  |
| 6. Relationship established | Was a relationship established prior to study commencement? | N/A (EDM: no previous contacts, MP: previous consultations with some patients) |
| 7. Participant knowledge of the interviewer | What did the participants know about the researcher? e.g. personal goals, reasons for doing the research | N/A (Interviewers’ background and research area were explained before the interviews) |
| 8. Interviewer characteristics | What characteristics were reported about the interviewer/facilitator? e.g. Bias, assumptions, reasons and interests in the research topic | N/A (Reasons and interests in the research topic were communicated) |

| **Domain 2: study design** |  |  |
| --- | --- | --- |
| *Theoretical framework* |  |  |
| 9. Methodological orientation and Theory | What methodological orientation was stated to underpin the study? e.g. grounded theory, discourse analysis, ethnography, phenomenology, content analysis | Page 6 |
| *Participant selection* |  |  |
| 10. Sampling | How were participants selected? e.g. purposive, convenience, consecutive, snowball | Page 5 |
| 11. Method of approach | How were participants approached? e.g. face-to-face, telephone, mail, email | Page 5 |
| 12. Sample size | How many participants were in the study? | Page 6 |
| 13. Non-participation | How many people refused to participate or dropped out? Reasons? | N/A (information not available) |
| *Setting* |  |  |
| 14. Setting of data collection | Where was the data collected? e.g. home, clinic, workplace | Page 6 |
| 15. Presence of non-participants | Was anyone else present besides the participants and researchers? | Page 6 |
| 16. Description of sample | What are the important characteristics of the sample? e.g. demographic data, date | Table 1 |
| *Data collection* |  |  |
| 17. Interview guide | Were questions, prompts, guides provided by the authors? Was it pilot tested? | Pages 5-6, Supplementary Data S2, Supplementary Data S3 |
| 18. Repeat interviews | Were repeat interviews carried out? If yes, how many? | N/A (No) |
| 19. Audio/visual recording | Did the research use audio or visual recording to collect the data? | Page 6 |
| 20. Field notes | Were ﬁeld notes made during and/or after the interview or focus group? | Page 6 |
| 21. Duration | What was the duration of the interviews or focus group? | Page 7 |
| 22. Data saturation | Was data saturation discussed? | Page 6 |
| 23. Transcripts returned | Were transcripts returned to participants for comment and/or correction? | N/A (No) |
| **Domain 3: analysis and ﬁndings** |  |  |
| *Data analysis* |  |  |
| 24. Number of data coders | How many data coders coded the data? | Page 6 |
| 25. Description of the coding tree | Did authors provide a description of the coding tree? | N/A (No) |
| 26. Derivation of themes | Were themes identiﬁed in advance or derived from the data? | Page 6 |
| 27. Software | What software, if applicable, was used to manage the data? | Page 6 |
| 28. Participant checking | Did participants provide feedback on the ﬁndings? | N/A (No) |
| *Reporting* |  |  |
| 29. Quotations presented | Were participant quotations presented to illustrate the themes/ﬁndings? Was each quotation identiﬁed? e.g. participant number | Pages 7-14 |
| 30. Data and ﬁndings consistent | Was there consistency between the data presented and the ﬁndings? | Pages 7-15 |
| 31. Clarity of major themes | Were major themes clearly presented in the ﬁndings? | Pages 7-14, Figure 1 |
| 32. Clarity of minor themes | Is there a description of diverse cases or discussion of minor themes? | Pages 7-14, Figure 1 |

**Supplementary Data S2:
Original semi-structured interview guide**

*I. INTRODUCTORY QUESTIONS*

1. How would you introduce yourself?

a. What is your family situation?

b. What is your job occupation, or what has been your occupation?

c. What are your hobbies?

*II. MAIN QUESTIONS*

2. How have you experienced living with rheumatoid arthritis so far?

a. How have you experienced your treatments?

b. What constitutes well-controlled rheumatoid arthritis for you?

c. How is your rheumatoid arthritis doing at present?

3. What complaints related to your rheumatoid arthritis do you still have at present?

4. What do you think is the cause of these complaints?

5. How do these complaints affect your life?

6. How do you act on these complaints? How do you manage these symptoms?

7. To what extent do you feel that your rheumatologist understands these complaints?

a. To what extent do you feel that your rheumatologist's assessment of the control of your rheumatoid arthritis is consistent with your own assessment?

8. To what extent do you think a change in your medication would relieve the symptoms?

a. What other things do you think you might still need?

9. Have other healthcare providers such as nurses, physiotherapists, occupational therapists or psychologists already counseled you for these symptoms? How have they affected your treatment?

a. To what extent do you think they could help with your complaints?

b. To what extent would you like them to be involved in your treatment?

*III. END*

10. Are there any particular issues that are important to you that have not yet been addressed in this interview?

11. How did you experience this interview? Were there any issues which you found difficult to talk about in this interview?

**Supplementary Data S3:
Revised semi-structured interview guide**

*I. INTRODUCTORY QUESTIONS*

1. How would you introduce yourself?

a. What is your family situation?

b. What is your job occupation, or what has been your occupation?

c. What are your hobbies?

*II. MAIN QUESTIONS*

2. How have you experienced living with rheumatoid arthritis so far?

a. How have you experienced your treatments?

b. What constitutes well-controlled rheumatoid arthritis for you?

c. How is your rheumatoid arthritis doing at present?

3. What complaints related to your rheumatoid arthritis do you still have at present?

4. How do these complaints affect your life?

5. What do you think is the cause of these complaints?

6. How do you act on these complaints? How do you manage these symptoms?

7. To what extent do you feel that your rheumatologist understands these complaints?

8. To what extent do you think a change in your medication would relieve the symptoms?

a. What other things do you think you might still need?

9. Have other healthcare providers besides your rheumatologist already counseled you for these symptoms? What have they meant to your treatment?

a. What could a nurse/physiotherapist/psychologist/sexologist/occupational therapist/dietician/social worker mean to you at this time?

b. With which healthcare providers are you not familiar?

c. To what extent do you think other healthcare providers other than your rheumatologist should be involved in your future follow-up? Who should be part of this team?

d. What disadvantages could you think of concerning these additional consultations?

*III. END*

10. Are there any particular issues that are important to you that have not yet been addressed in this interview?

11. How did you experience this interview? Were there any issues which you found difficult to talk about in this interview?
